# Docosahexaenoic acid and disulfiram act in concert to kill cancer cells: a mutual enhancement of their anticancer actions

**DOI:** 10.18632/oncotarget.14702

**Published:** 2017-01-17

**Authors:** Yang Jiao, Bethany N. Hannafon, Roy R. Zhang, Kar-Ming Fung, Wei-Qun Ding

**Affiliations:** ^1^ Department of Pathology, University of Oklahoma Health Sciences Center, Oklahoma City, OK 73104, USA; ^2^ School of Radiation Medicine and Protection, Medical College of Soochow University, Suzhou 215123, P.R. China; ^3^ Peggy and Charles Stephenson Cancer Center, Oklahoma City, OK 73104, USA

**Keywords:** docosahexaenoic acid, disulfiram, oxidative stress, nuclear factor (erythroid-derived 2)-like 2, cancer stem cell

## Abstract

We previously reported a synergistic anticancer action of clioquinol and docosahexaenoic acid (DHA) in human cancer cells. However, clioquinol has been banned from the clinic due to its neurotoxicity. This study identified disulfiram (DSF) as a substitute compound to clioquinol, acting in concert with DHA to more effectively kill cancer cells and suppress tumor growth. Treatment with DSF and DHA induced greater apoptotic cell death and suppression of tumor growth *in vitro* and *in vivo*, as compared to DSF and DHA used alone. Mechanistic studies demonstrated that DSF enhances DHA-induced cellular oxidative stress as evidenced by up-regulation of Nrf2-mediated heme oxygenase 1 (HO-1) gene transcription. On the other hand, DHA was found to enhance DSF-induced suppression of mammosphere formation and stem cell frequency in a selected cancer model system, indicating that alterations to cancer cell stemness are involved in the combinatory anticancer action of DSF and DHA. Thus, DHA and DSF, both clinically approved drugs, act in concert to more effectively kill cancer cells. This combinatory action involves an enhancement of cellular oxidative stress and suppression of cancer cell stemness.

## INTRODUCTION

**Figure 1:**
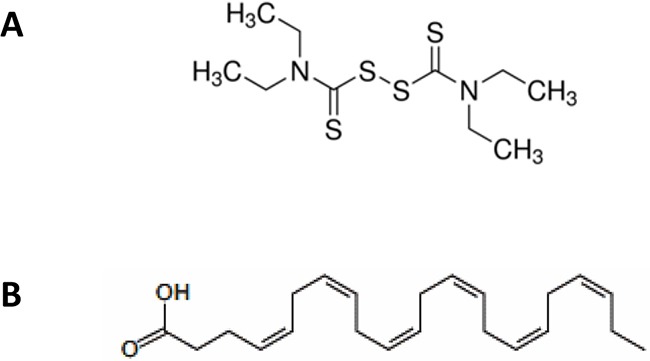
Molecular structure of disulfirm (DSF, A) and docosahexaenoic acid (DHA, B)

New drug combinations have been clinically and experimentally tested with the goal of improving cancer therapeutic efficacy and minimizing side-effects and drug resistance [1–4]. However, ideal combination therapies remain to be developed. In the course of searching for novel and effective combination therapies, we have previously reported that docosahexaenoic acid (DHA, 22:6, n-3), a long chain n-3 polyunsaturated fatty acid (n-3 PUFA, Figure 1), and clioquinol, a metal binding compound act in synergy to kill human cancer cells [5, 6].

The anticancer properties of DHA have been well characterized in various experimental model systems [2, 7–9]. DHA is an essential long-chain n-3 PUFA that is beneficial to the health of the cardiovascular and central nervous systems [10–14]. DHAs’ ability to selectively inhibit tumor cell viability while showing less toxicity towards normal cells, and its history of extensive and safe use in humans, justify DHA as a promising anticancer agent for combination therapy [2, 15]. Mechanistic studies have implied that lipid peroxidation induced by DHA within tumor cells plays a crucial role in its anticancer action [16–18]. On the other hand, clioquinol is an antibiotic and metal binding compound that was reported to have anticancer activity *in vitro* and *in vivo* [5, 19, 20]. Historically, clioquinol was clinically used for treating diarrhea and skin infection. Unfortunately, it has been banned clinically in many countries because of its observed neurological toxicity [21, 22]. Due to this limitation, we sought to find alternative compounds to clioquinol that may act in concert with DHA to more effectively kill cancer cells and suppress tumor progression.

DSF, a derivative of thiuram and a metal binding compound, has been safely used to combat alcoholism in human for more than sixty years with well characterized pharmacodynamics and kinetics (Figure 1) [23, 24]. Recently, its potential as an anticancer drug and as an adjuvant therapy has been tested both in preclinical studies and clinical trials [23, 25–27]. The mechanisms of DSF's anticancer effects are versatile. For example, serving as a metal binding compound of cellular heavy metal ions, similar to clioquinol, DSF impairs the activities of zinc- or copper-dependent enzymes, such as superoxide dismutases, matrix metalloproteinases, and inhibits proteasome activity, leading to inhibition of tumor angiogenesis, cancer cell invasion, and metastasis [23, 26, 28]. Interestingly DSF is considered as a metal ionophore [26, 28], a feature that has been well recognized for clioquinol [19, 29], In addition, DSF has long been recognized for its inhibitory activity against aldehyde dehydrogenase (ALDH), an enzyme having the strongest association with the cancer stem cell (CSC) phenotype [23]. Inhibition of ALDH activity by DSF has been reported to play a key role in suppressing the growth of CSCs derived from cancers of the brain, breast, ovary, pancreas, lung, liver, and blood [30–35]. Because DSF and clioquinol display similarity in metal ion binding and cellular sequestration [26, 28], we envisioned that DSF could serve as an excellent alternative compound to clioquinol, that can be tested for combination therapy with DHA.

We report here that DHA and DSF act in concert to more effectively kill cancer cells and suppress tumor progression both *in vitro* and *in vivo*. Our results suggest that this anticancer action is mediated in part through enhancing cellular oxidative stress and suppressing cancer cell stemness.

## RESULTS

### DSF and DHA work together to more effectively induce apoptosis and suppress cancer cell growth

**Figure 2:**
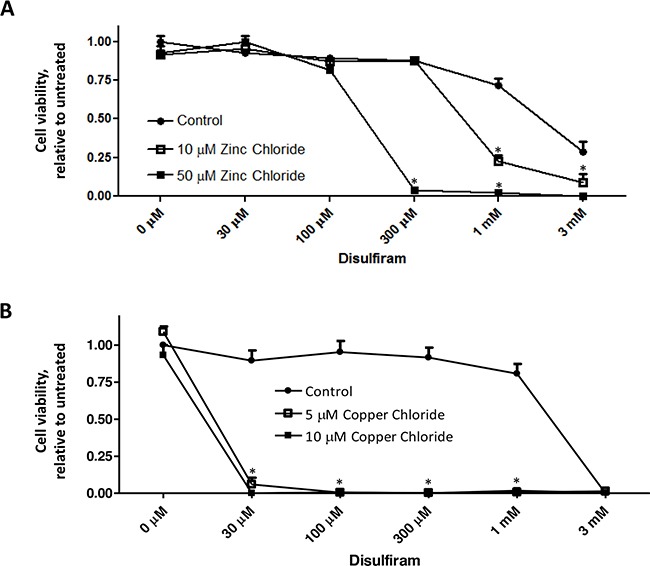
Metal ions enhance DSF-induced suppression of cancer cell viability MDA-MB-231 cells were treated with DSF, in the presence or absence of zinc chloride **A**. or copper chloride **B**. at the indicated concentrations for 72 h. MTS assay was performed, and the representative result of three independent experiments was shown. Cell viability was expressed relative to untreated control cells. * P < 0.05, compared to untreated cells using one-way ANOVA.

DSF (Figure 1) and its metabolites can form strong complexes with endogenous heavy metal ions such as copper or zinc, resulting in inhibited activities of zinc- and copper-dependent enzymes (such as superoxide dismutase, matrix metalloproteinase, etc.), and in turn elevating cellular oxidative stress or impeding cancer cell invasion, angiogenesis, or metastasis [26, 36–38]. In the present study, we first confirmed that DSF's cytotoxicity was dramatically enhanced by copper or zinc ions. DSF alone exhibited its cytotoxicity on the human breast cancer cell line MDA-MB-231 at a relatively high concentration (> 1mM), whereas its cytotoxicity was significantly increased in the presence of various concentrations of copper and zinc ions (P<0.05) (Figure 2). These results are in accordance with the characteristic of DSF as a metal binding compound, similar to that of clioquinol [19, 39]. The enhanced cytotoxicity of DSF plus copper or zinc was also evident in several other human cancer cell lines (data not shown), including A2780 (ovarian), BT-20 and MCF7 (breast), suggesting that this is not a cell line specific effect.

**Figure 3:**
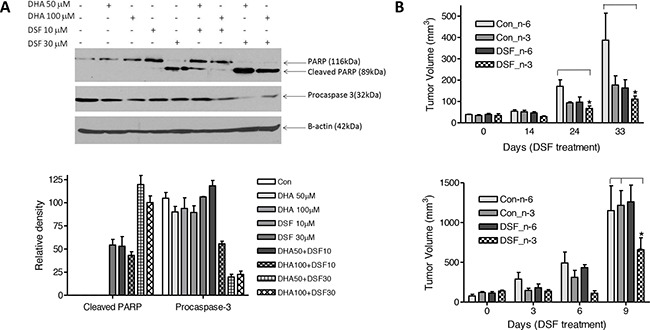
DSF and DHA work together to induce apoptosis and suppress tumor growth **A**. MDA-MB-231 cells were incubated with DHA and/or DSF at indicated concentrations for 24 h. Cell lysates were collected, and protein expression of PARP, procaspase-3, and β-actin was analyzed by Western blot assay (**top**). Expression of procaspase-3 and the cleaved PARP was quantified and normalized to β-actin (**low**, n=3). The data are expressed as percent of control group (procaspase-3) or percent of DHA 100 μM plus DSF 30 μM group (cleaved PARP). **B**. MDA-MB-231 cells (10^6^/0.l mL, less aggressive model, **top**) were inoculated into the left flank of 5-week-old female nude mice. The mice were fed with 7.5% corn oil diet (high n-6 PUFA content) or 7.5% fish oil diet (high n-3 PUFA content). Three weeks later, the mice were randomized into 4 groups and were treated with solvent or DSF (75 mg/.kg) for 4 therapeutic cycles. For an aggressive model, MDA-MB-231 cells (5×10^6^/0.l mL, **low**) were inoculated into the left flank of 5-week-old female nude mice. The mice were fed with 7.5% corn oil diet (high n-6 PUFA content) or 7.5% fish oil diet (high n-3 PUFA content). One week later, the mice were randomized into 4 groups and were treated with solvent or DSF (75 mg/kg) for 5 days. Tumor volume was measured and calculated by the following formula: V=1/2(A×B^2^), where V is the tumor volume, A is the length, and B is the width of the tumor (n=5). *,P<0.05, using Two-way ANOVA.

The combination of DHA and clioquinol has been shown to significantly promote apoptotic death of human cancer cells [5]. To determine whether treatment with DSF and DHA enhances apoptosis of cancer cells, MDA-MB-231 cells were exposed to 50-100μM DHA in the presence or absence of 10-30μM DSF for 24 h. PARP cleavage and procaspase 3 detection were measured for detection of apoptosis, as we previously described [5, 19]. Figure 3A shows that the combination of DSF and DHA significantly enhances PARP cleavage and caspase-3 activation (shown by attenuated procaspase-3 expression), results that are supportive of our hypothesis. To further affirm the combinatory effect of DHA and DSF on cancer growth, MDA-MB-231 cells were implanted to the flanks of nude mice using both aggressive (5×10^6^ cells) and less aggressive (1×10^6^ cells) models. The mice were fed a corn oil-based diet (7.5%, wt/wt, high n-6 PUFAs) or a fish oil-based diet (7.5%, wt/wt, high n-3 PUFA (DHA), Table 1) [40]. DSF was delivered through I.P. at 75mg/kg. No significant difference of the body weight was observed among different groups of mice (data not shown). However, as shown in Figure 3B, as early as 14 days after DSF treatment in the less aggressive model, the tumor growth was significantly suppressed in mice fed with a fish oil-based diet compared to mice fed a corn oil-based diet (P<0.05). The significant suppression of tumor growth in fish oil fed mice was evident after 24 days of DSF treatment, and the trend lasted until the end of the experiment (Figure 3B, top). Furthermore, in the aggressive model, the tumor growth was significantly suppressed by DSF after 9 days of treatment in mice fed a fish oil diet as compared to mice fed a fish oil diet alone (P<0.05), consistent with the observations from the less aggressive model.

**Table 1:** Composition of corn oil and fish oil diets (g/Kg)

Diet ingredients	Fish oil	Corn oil
Casein	200.0	200.0
DL-Methionine	3.0	3.0
Sucrose	475.0	475.0
Corn Starch	150.0	150.0
Fish oil	75.0	0.0
Corn oil	0.0	75.0
Cellulose	50.0	50.0
Mineral Mix, AIN-76	35.0	35.0
Vitamin Mix, AIN-76A	10.0	10.0
Choline Bitartrate	2.0	2.0
Ethoxyquin, antioxidant	0.015	0.015
Yellow food color	0.1	0.1

Based on our previous experience with clioquinol and DHA [6], we assumed that the enhanced anticancer action by the combination of DSF and DHA would not be cell line specific. In fact the cytotoxic effects of the combination of DSF and DHA were also evident in A2780 and BT-20 cells (data now shown).

### DSF enhances DHA-induced HO-1 gene transcription

**Figure 4:**
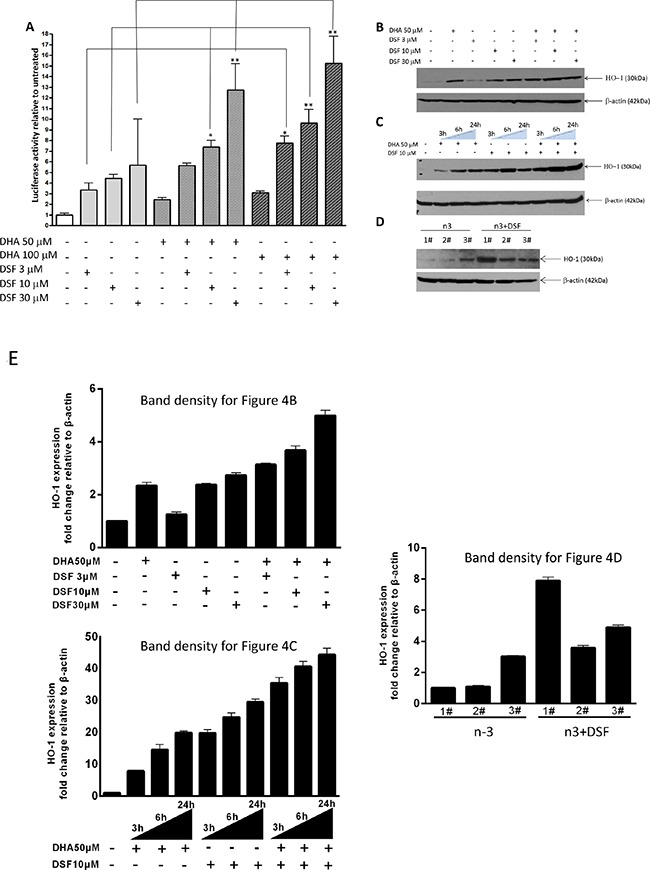
DSF enhances DHA-induced HO-1 gene transcription **A**. MDA-MB-231 cells were transfected with the PGL3/HO-1-promoter construct and treated with DHA (50μM or 100μM) in the presence or absence of DSF (3μM, 10μM, 30μM) for 21 h. Luciferase activity was analyzed in the cell lysate and is expressed relative to the levels in untreated cells. *, P<0.05, **, P<0.01, versus untreated control cells using one-way ANOVA followed by Dunnett's analysis (n=4). **B**. MDA-MB-231 cells were treated with DSF (3μM, 10μM, 30μM) with or without 50μM DHA for 24 h, or **C**. treated with 50μM DHA and 10μM DSF for 3, 6, or 24 h. HO-1 expression was detected by Western blot. β-actin served as a loading control. **D**. HO-1 expression was detected by Western blot in tumor tissues from xenograft mice fed a fish oil diet (n-3) and treated with DSF (75mg/kg). β-actin served as a loading control. **E**. Expression of HO-1, detected in B, C and D, was quantified and normalized to β-actin.

**Figure 5:**
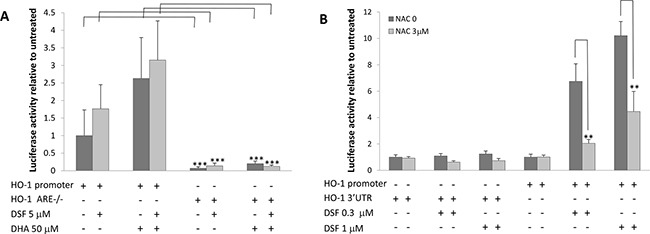
DSF enhances DHA-induced HO-1 gene transcription **A**. A2780 cells were transfected with the PGL3/HO-1-promoter or ARE double deleted HO-1 promoter constructs for 24 h. The cells were then treated with 50μM DHA and 5μM DSF for 21 h. The luciferase activity was analyzed (n=4). ***, P<0.001, Student's t-test. **B**. A2780 cells were transfected with the PGL3/HO-1-promoter or PGL3/HO-1 3’UTR constructs for 24 h, and treated with 3μM NAC for 1 h followed by DSF (0.3μM or 1μM) for additional 21 h. The luciferase activity was analyzed (n=4). **, P<0.01, Student's t-test.

We and others have previously demonstrated that DHA-induced lipid peroxidation in cancer cells is primarily accountable for DHA's anticancer activity [41]. We showed that in responding to DHA-induced lipid peroxidation, the expression of heme oxygenase 1 (HO-1), a crucial cytoprotective antioxidant enzyme, is highly induced [40]. We therefore envisioned that the combination of DHA and DSF may enhance DHA's anticancer activity through augmenting DHA-induced oxidative stress. Indeed, we found that the HO-1 expression is significantly enhanced in cells that were treated with the combination of DHA (50-100μM) and DSF (1-3μM) as compared to cells treated with either compounds alone. This was analyzed by the reporter gene assay on HO-1 gene promoter activity (Figure 4A) and by western blot on HO-1 protein levels (Figure 4B, 4C). The HO-1 protein expression was enhanced by the combination of DHA and DSF in a concentration- and time-dependent manner. Interestingly, DSF alone seemed able to induce HO-1 expression, an observation that has not been previously reported. Furthermore, an increased HO-1 protein expression was detected in xenograft tissues of the mice that were fed a fish oil-based diet and treated with DSF as compared to the mice without DSF treatment (Figure 4D). Taken together, both the *in vitro* and the *in vivo* results indicated that the combination of DSF and DHA enhances HO-1 expression in cancer cells. We have previously demonstrated that DHA-induced HO-1 gene transcription in A2780 cells is mainly regulated by the Nrf2 antioxidant pathway that targets the antioxidant responsive elements (ARE) localized in the HO-1 promoter region [40]. To determine whether this signaling mechanism also mediates DSF enhancement of the DHA-induced HO-1 expression, the HO-1 promoter reporter gene constructs, with or without deletion of the AREs, were transfected to A2780 cells. As shown in Figure 5A, deletion of the two AREs in the HO-1 gene promoter completely abolishes DHA-induced HO-1 gene transcription, regardless of the presence or absence of DSF, indicating the critical involvement of the Nrf2-ARE signaling in this event. The fact that DSF-induced HO-1 gene promoter activity was also abolished by the deletion of AREs suggested that oxidative stress is responsible for DSF-induced HO-1 gene expression. This was confirmed by the use of N-Acetyl Cysteine (NAC), an antioxidant reagent, which attenuated DSF-induced HO-1 gene transcription (P<0.01, Figure 5B). Both NAC and DSF failed to affect HO-1 3’UTR-mediated luciferase activity, verifying that it is the HO-1 gene transcription that is affected by DSF. Together, these results, along with our previous reports [40], suggested that the HO-1 induction by DSF and DHA is mediated by oxidative stress targeting the Nrf2-ARE signaling pathway.

### DHA enhances DSF-induced suppression of cancer cell stemness

**Figure 6:**
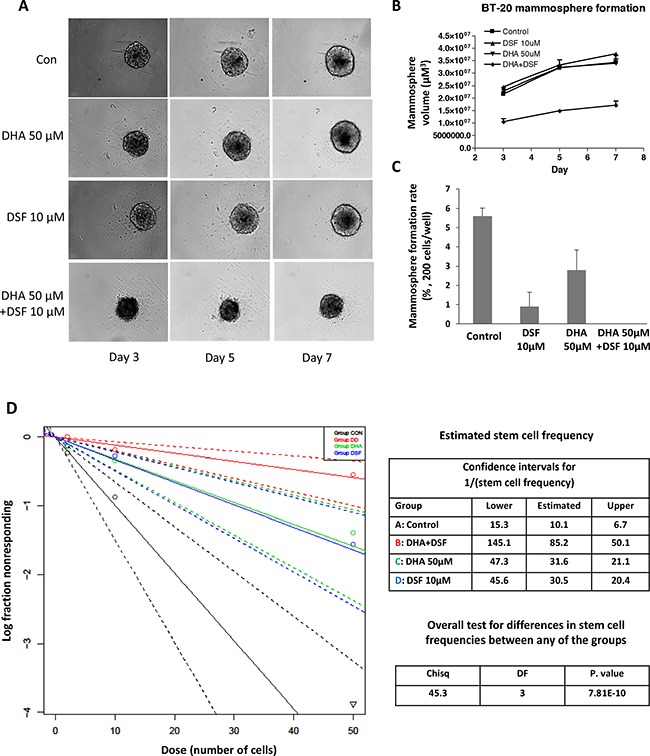
DHA enhances DSF-induced suppression of mammosphere formation BT-20 cells were seeded onto 96-well ultra-low attachment plates in sphere culture serum free media, and treated with 50μM DHA and 10μM DSF as described in the methods section. Mammosphere formation was observed and photographed using the PerkinElmer Operetta system **A**. The volume **B**. and formation rate **C**. of mammosphere were analyzed. *, P<0.05 versus untreated control using one-way ANOVA followed by Dunnett's analysis. **D**. The *In vitro* Extreme Limiting Dilution assay was performed 48 h after DHA and DSF treatment. Cells were plated into 96-well ultra-low attachment plates with various seeding densities (0.4–50 cells/well in 200 μL) and were cultured until day 14 at 37°C. At the time of quantification, each well was examined for the formation of mammospheres, and data were analyzed using extreme limiting dilution software web interface.

DSF and its cellular metabolites have been well characterized for their inhibitory activity on ALDH [24]. Meanwhile ALDH has been generally accepted as a cancer stem cell (CSC) marker [42]. Accordingly, DSF's anticancer activity is considered to be associated with its inhibitory effect on cancer cell stemness among various types of malignancies [23, 30–33, 35]. In the present study, mammosphere formation [43] and *in vitro* Extreme Limiting Dilution (ELDA) assay [44, 45] were applied to determine whether DHA enhances DSF's suppression of cancer cell stemness. The human breast cancer cell line BT-20 was used for mammosphere formation in this study. Treatment with 10μM DSF or 50 μM DHA alone minimally affected the appearance of the already formed mammospheres (Figure 6A); however the combination of DSF and DHA significantly decreased the mammosphere volume (Figure 6A, 6B). Furthermore, pretreatment with DSF alone for 48 hours significantly prevented mammosphere formation by reducing the mammosphere formation rate, indicating its ability to suppress cancel cell stemness (Figure 6C). When the cells were pre-treated with DSF plus DHA, the formation rate of BT-20 mammospheres was completely depleted (Figure 6C). The *in vitro* ELDA assay was performed to confirm the effects of DSF and/or DHA on the cancer stem cell frequency. As shown in Figure 6D, a significant decrease in the cancer stem cell frequency was observed in cells pretreated with DSF and DHA, compared to those pretreated with each compound alone (P<0.001). The cancer stem cell frequency was 1/10.1 in control cells, 1/85 in DSF/DHA-pretreated cells, 1/30.5 in DSF-pretreated cells, and 1/31.6 in DHA-pretreated cells. Taken together, these results illustrated that the suppression of cancer cell stemness by DSF is further enhanced by DHA, which offered another cellular mechanism to explain the enhanced anticancer activity by the combination of DSF and DHA.

## DISCUSSION

We have previously demonstrated the synergistic anticancer actions of clioquinol and DHA [5]. However, further development of this combination therapy is impeded by the fact that cliquinol is neurotoxic and has been banned from clinical use in many countries [46–48]. We sought to find a substitute compound that may work with DHA as a potential combination cancer therapy. In this context, the most interesting finding from the present study is that we have identified DSF, a metal binding compound similar to clioquinol, that acts in concert with DHA to more effectively kill cancer cells. Because DSF has been used in humans for many years for the treatment of alcoholism [24], and DHA is a major component of fish oil supplements [49] and a prescribed drug [50], the combination of these two could be a promising strategy for more safe and effective cancer treatment.

We first confirmed that DSF acts as a metal binding compound to suppress cancer cell viability. DSF‘s cytotoxicity was dramatically enhanced by addition of copper or zinc ions indicating its similarity to clioquinol, belonging to a group of anticancer compounds, namely metal ionophores [29]. We then tested the anticancer action of DSF plus DHA in different cancer model systems. Our experiments provide evidence to demonstrate a more effective anticancer action when DSF and DHA are used in combination. First, we show that DSF plus DHA more effectively induce apoptosis of human cancer cells, as evidenced by PARP cleavage and caspase 3 activation. These observations indicate that the combination of DSF and DHA can more effectively kill human cancer cells. Second, in a xenograft nude mouse model, we demonstrated that the combination of DSF treatment and a fish oil diet containing high DHA content significantly suppresses tumor growth as compared to mice with corn oil diet, thus providing *in vivo* evidence of an enhanced anticancer activity when DSF is combined with DHA. Note that the body weight is similar among groups of mice, suggesting that the combination of DSF and DHA is tolerable *in vivo*. The selective action of DSF with the fish oil diet versus corn oil diet is consistent with previous conclusions that n-3 PUFA, but not n-6 PUFA, suppresses tumor growth in various cancer model systems [51, 52]. Together, these observations clearly demonstrate that the combination of DSF and DHA is more effective in killing cancer cells and slowing down tumor growth.

In our search for potential cellular mechanisms behind the enhanced cytotoxicity of the combination of DSF and DHA, we assumed that either DHA's anticancer action is enhanced by DSF, or vice versa, that DSF's anticancer activity is enhanced by DHA. We discovered that the combination of DHA and DSF indeed leads to a mutual enhancement of their actions in cancer cells, including DHA-induced oxidative response, and DSF-induced suppression of mammosphere formation.

In our previous studies, we demonstrated that lipid peroxidation is primarily responsible for DHA-induced apoptotic cancer cell death [53]. Several studies also showed that the tumor suppressive effects of DHA may be accelerated by increased cellular oxidative stress [2, 17, 18, 54]. Coincidentally, DSF and its metabolites have been shown repeatedly to cause a pro-oxidative environment in cancer cells [17, 23, 26, 36]. Therefore, it is not surprising that the combination of DSF and DHA causes significantly higher oxidative pressure as indicated by enhanced HO-1 expression. Indeed, DSF alone could induce HO-1 transcription which can be attenuated by the antioxidant NAC, results consistent with the concept that DSF induces a pro-oxidative environment in cancer cells. Furthermore, in the absence of AREs in the HO-1 gene promoter, both DHA- and DSF-induced HO-1 gene promoter activity was abolished, further indicating that the Nrf2-ARE signaling, a well-established signaling pathway mediating cellular oxidative stress, is essential for this event. Thus, our results clearly indicate that cellular oxidative stress is enhanced by the combination of DSF and DHA which could lead to more effective killing of cancer cells

On the other hand, it has long been established that DSF has inhibitory activity against ALDH, specifically ALDH1A1 [24], and ALDH activity is highly associated with cancer cell stemness [42]. Therefore, the suppression of ALDH activity by DSF was thought to play a key role in its anticancer action [23]. In fact, DSF-induced suppression of CSCs has been extensively studied and well-recognized [23]. Meanwhile, *in vitro* and/or *in vivo* studies have confirmed that DHA also possesses the potential to eliminate CSCs [55–58]. Therefore, mammosphere formation and ELDA assay were applied to test whether DSF's inhibitory action on CSCs is enhanced by DHA.

It is well-known that cells derived from mammary tumors tend to form mammospheres under anchorage-independent conditions [59]. In the present study, although pre-formed mammospheres seemed to be unaffected by either DSF or DHA treatment, DSF pretreatment alone significantly suppressed the mammosphere formation rate, and the combined treatment of DSF and DHA further decreased both the mammosphere size and the formation rate, strongly indicating that the combination is more effective in suppressing cancer cell stemness. Theoretically, a single CSC is sufficient to form a mammosphere [44, 60]; therefore the number of mammospheres after treatment represents the number of CSCs. We used the ELDA assay to statistically analyze the number of CSCs in each treatment regimen (DSF alone, DHA alone, and DSF/DHA combination) according to a Poisson distribution. The results demonstrate that the CSC frequency in both DSF and DHA treated breast cancer cells are reduced. A further reduction of the CSC frequency was observed in BT-20 cells pretreated with DSF and DHA. These data demonstrate that the combination of DHA and DSF suppresses mammosphere formation and stem cell frequency in a breast cancer model system, which could in part account for their combinatory anticancer action.

In summary, we have demonstrated that DHA and DSF act in concert to more effectively kill cancer cells and suppress tumor progression. The potential mechanisms behind their combinatory anticancer action include an enhancement of cellular oxidative stress and the suppression of cancer cell stemness. To our knowledge, this is the first attempt to explore the combined anticancer properties of these two compounds. Because both DSF and DHA are approved by the Food and Drug Administration for human use, further investigation on the potential of this combination therapy in clinical practice is warranted.

## MATERIALS AND METHODS

### Cell culture and chemical compounds

Human ovarian cancer cell line A2780, breast cancer cell lines MDA-MB-231 and BT-20 were purchased from American Type Culture Collection (ATCC, Manassas, VA, USA). A2780 cells were maintained in RPMI 1640 medium, while the breast cancer cell lines were cultured with DMEM medium, respectively. Growth medium was supplemented with 10% fetal bovine serum, 100 units/ml penicillin, and 100 units/ml streptomycin (Life Technologies Inc, Grand Island, NY, USA). Cells were cultured in an incubator with 5% CO_2_ at 37°C, and sub-cultured every other day. Analytic grade DSF, DHA, N-Acetyl-Cysteine (NAC), copper chloride, and zinc chloride were purchased from Sigma-Aldrich (St. Louis, MO, USA). The stock solution of DSF, DHA, NAC, copper chloride and zinc chloride was prepared in corresponding solvent as previously described [37, 61].

### MTS cell viability assay

Exponential growth cancer cells were seeded onto 96-well plate at a density of 5,500/well. Twenty-four hours following seeding, cells were treated with various compounds as previously described [40]. The cells were cultured at 37°C with 5% CO_2_ for additional 72 h after initiation of the treatment. For each well, the attached cells were incubated in 100μL growth medium supplemented with 20μL CellTiter 96® AQueous One Solution (Promega, Madison, WI, USA) and incubated for 1 h. The absorbance value at 495nm was recorded using a spectrometer. The cell viability was calculated using the following formula: OD 490nm of treatment group/OD 490nm of control group × 100%.

### Western blot assay

For whole cell lysate preparation, cells were mechanically detached from culture plates and collected by centrifugation at 3,000rpm for 5 min at 4 °C, lysed in RIPA lysis buffer supplemented with the proteinase inhibitor cocktail (Roche, Indianapolis, IN, USA), and centrifuged at 13,000rpm to collect the supernatants. For nuclear protein extraction, cells were incubated with 2mL wash buffer (1mM HEPES pH7.9, 0.15mM MgCl_2_, 1mM KCl, 0.05mM Dithiothreitol, 0.01% NP-40, 1 × proteinase inhibitor cocktail) on ice for 5 min, and proceeded to centrifugation at 4000rpm for 5 min. The supernatant was removed, and the cell pellet was incubated on ice for 30 min with 50μL suspension buffer (25% glycerol, 0.42M NaCl, 1.5mM MgCl_2_, 0.2mM EDTA, 0.5mM DTT, 1×proteinase inhibitor cocktail), followed by centrifugation at 13000rpm for 20 min for nuclear protein collection. Western blot was performed as previously described [40]. In brief, 30 μg proteins of each sample were separated by SDS-PAGE and transferred onto PVDF membranes (EMD Millipore, Billerica, MA, USA) under appropriate condition. Following blocking procedure by 5% non-fat milk, PVDF membranes were subsequently incubated in primary antibody solution, secondary antibody solution, washed, and finally proceeded to chemiluminescence (Thermo Scientific, Rockford, IL, USA) and X-ray film exposure (Denville Scientific Inc., Holliston, MA, USA). The primary antibodies used in this study were as follows: anti-PARP (1:1000, Cell Signaling Technology, Beverly, MA, USA), anti-Procaspase 3 (1:200, 31A1067, Santa Cruz Bio Technology Inc., Dallas, Texas, USA), anti-HO-1 (1:2000, Enzo Life Sciences, Farmingdale, NY, USA), anti-β-Actin (1:5000, Sigma-Aldrich, St. Louis, MO, USA). The secondary antibodies were goat-anti-mouse IgG-HRP, goat-anti-rabbit IgG-HRP (1:5000, Santa Cruz Bio Technology Inc., Dallas, Texas, USA). Protein expression of Procaspase 3, cleaved PARP, and HO-1, was semi-quantified by densitometry using Adobe Photoshop Elements 6.0 (San Jose, CA) and normalized to that of β-actin.

### Human breast cancer xenograft mice experiment

Five-week-old Balb/C nude mice were purchased from Taconic Farms Inc. (Germantown, NY) and used for the *in vivo* study. The research protocol was in accordance with the Institute Animal Care and Use Committee procedures and guidelines. One week before breast cancer cells inoculation, the mice were randomly divided into 7.5% fish oil diet and 7.5% corn oil diet (Teklad Diets, Madison, WI) groups, and fed with the assigned diet during the whole procedure. The human breast cancer cell line MDA-MB-231 was inoculated on the left flanks of nude mice at density of 10^6^/0.1mL PBS-Matrigel for a less aggressive model, and 5×10^6^/0.1mL PBS-Matrigel for an aggressive model. Three weeks (less aggressive model) or one week (aggressive model) after inoculation for each diet group the mice were randomly divided into two subgroups (day 0), which were treated with DSF (75mg/kg) or vehicle (PBS/Cremophor/DMSO=7.5/2/0.5) through I.P.. A 5-day consecutive daily injection was regarded as a treatment cycle, and all the mice in the less aggressive model were underwent 4 complete treatment cycles. One treatment cycle was completed for the aggressive model. Tumor volume was measured using the following formula: V=1/2(A×B^2^), where V is the tumor volume, A is the length, and B is the width of the xenograft. The tumor volume and body weight were recorded 3 times a week. At the end of the experiment, the mice were euthanized. The tumors were excised and prepared for Western blot assay.

### Dual-luciferase reporter gene assay

Dual-luciferase reporter gene assay was performed as previously described [40]. Briefly, cells were seeded onto 100mm culture plates at a density of 2.2 × 10^6^ and cultured overnight. The luciferase reporter constructs including the pGL3/4.5-HO-1 promoter, pGL3/4.5-HO-1 ARE mutants, and the HO-1-3’-UTR were applied for transfection using the Fugene HD transfection reagent (Roche, Indianapolis, IN, USA) [40]. Twenty-four hours after transfection, cells were re-plated into 96-well plates at a density of 1×10^5^ per well. The following day, cells were treated with various compounds at indicated concentrations and durations. Luciferase activity was assayed using the Dual-Luciferase Reporter kit (Promega, Madison, WI, USA), according to the manufacture's instruction. The data was calculated as firefly/renilla for each sample and luciferase activity was presented as arbitrary units relative to untreated control cells.

### Mammosphere formation assay

In order to determine the effects of DSF and/or DHA on the mammosphere formation ability, a BT-20 single cell suspension was cultured in 96-well ultra-low adherence plates (Perkin Elmer, Waltham, MA, USA) at a density of 2,000 cells/ml with the stem cell medium, which was made of serum-free DMEM:F12 medium supplemented with B27 (Life Technologies Inc., Grand Island, NY, USA), 10ng/ml basic fibroblasts growth factor, 5 μg/ml insulin, 0.4% BSA (Sigma-Aldrich, St. Louis, MO, USA), 2ng/ml epidermal growth factor (Pepro Tech Inc., Rocky Hill, NJ, USA), and 100U/ml antibiotics (Life Technologies Inc., Grand Island, NY, USA). Five days after seeding, the mammospheres were exposed to drugs and cultured for another 7 days. The size of mammosphere was monitored and calculated every other day after drug treatment, using an Operetta high content imaging system (Perkin Elmer, Waltham, MA, USA). For mammosphere formation rate detection, BT-20 cells were pretreated with drugs for 48 h, and then were sub-cultured in 96-well ultra-low adherence plates as described above. The mammospheres with the diameter greater than 40μm were counted under microscope 14 days after subculture. The mammosphere formation rate was presented as percentages of numbers of mammosphere/cells seeded.

### *In vitro* extreme limiting dilution assay

The stem cell frequency was calculated by *in vitro* extreme limiting dilution assay (ELDA). Exponential growing BT-20 cells were pretreated with drugs for 48 h, dissociated into single-cell suspensions, and were plated into 96-well plates in sphere culture media with various seeding densities (0.4–50 cells/well in 200μL). The spheres were cultured for 10 days at 37 °C, and quantified for the frequencies of mammosphere formation at the end of the assay, using extreme limiting dilution software web interface (http://bioinf.wehi.edu.au/software/elda/).

### Statistics

Statistical analysis was done with Graphpad Prism software (San Diego, CA, USA). Differences among control and experimental groups were determined by two tailed T test or one-way ANOVA with Dunnett's post-test, with p < 0.05 or p < 0.01 as the level of statistical significance.

## Abbreviations

ALDH: aldehyde dehydrogenase
ARE: antioxidant response element
CSC: cancer stem cells
DHA: docosahexaenoic acid
DSF: disulfiram
HO-1: heme oxygenase 1
NAC: N-Acetyl-Cysteine
Nrf2: nuclear factor (erythroid-derived 2)-like 2
PUFA: polyunsaturated fatty acid
